# Acute progesterone feedback on gonadotropin secretion is not demonstrably altered in estradiol‐pretreated women with polycystic ovary syndrome

**DOI:** 10.14814/phy2.15233

**Published:** 2022-04-05

**Authors:** Su Hee Kim, Jessica A. Lundgren, James T. Patrie, Christine M. Burt Solorzano, Christopher R. McCartney

**Affiliations:** ^1^ Center for Research in Reproduction University of Virginia School of Medicine Charlottesville Virginia USA; ^2^ Division of Endocrinology Department of Medicine University of Virginia School of Medicine Charlottesville Virginia USA; ^3^ Department of Public Health Sciences University of Virginia School of Medicine Charlottesville Virginia USA; ^4^ Division of Endocrinology Department of Pediatrics University of Virginia School of Medicine Charlottesville Virginia USA

**Keywords:** estradiol, FSH, LH, PCOS, progesterone

## Abstract

Women with polycystic ovary syndrome (PCOS) demonstrate gonadotropin‐releasing hormone (GnRH) pulse generator resistance to suppression with 7 days of progesterone and estradiol administration. It remains unknown whether such women demonstrate impairments in *acute* progesterone negative feedback on LH pulse frequency or progesterone *positive* feedback on gonadotropin release. This was a randomized, double‐blind, placebo‐controlled crossover study designed to test the hypothesis that acute, progesterone‐related suppression of LH pulse frequency and progesterone‐related augmentation of gonadotropin release are impaired in PCOS. Twelve normally cycling women and 12 women with PCOS completed study. Volunteers were pretreated with transdermal estradiol (0.2 mg/day) for 3 days and then underwent a frequent blood sampling study (20:00–20:00 h), during which they received micronized progesterone (100 mg) or placebo at 06:00 h. In a second study admission, volunteers received the intervention they did not receive during the first admission, but the protocol was otherwise identical. The primary outcome measures were LH secretory characteristics and circulating gonadotropin concentrations. Exogenous progesterone did not reduce LH pulse frequency in either group. Mean LH, pulsatile LH secretion, LH pulse mass, and mean FSH increased more with progesterone compared to placebo in both groups. Although trends toward less pronounced changes in LH pulse mass and pulsatile LH secretion were observed in the PCOS group, these differences were not statistically significant. In summary, exogenous progesterone did not suppress LH pulse frequency within 12 hours in estradiol‐pretreated women, and the positive feedback effect of progesterone on gonadotropin release was not demonstrably impaired in PCOS.

**New & Noteworthy:**

This study indicated that exogenous progesterone does not reduce LH pulse frequency within 12 h in women with PCOS, but progesterone acutely increased gonadotropin in these women. This study suggested that progesterone‐related augmentation of gonadotropin release may be impaired in PCOS compared to normally cycling women, but this finding was not statistically significant.

## Graphical abstract

This study indicated that exogenous progesterone does not acutely (within 12 hours) reduce LH pulse frequency in women with PCOS. This study also suggested that progesterone‐related augmentation of gonadotropin release is not demonstrably impaired in PCOS.

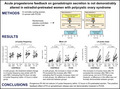

## 1 INTRODUCTION

Progesterone is the primary regulator of cyclic gonadotropin‐releasing hormone (GnRH) pulse frequency reduction in adult women (Soules et al., 1984), and a reduction in GnRH pulse frequency during the luteal phase appears to be important for normal long‐term cyclic function (Lam & Ferin, 1987; Soules et al., 1987). However, it remains unknown how rapidly progesterone suppresses GnRH pulse frequency in women. In cows and sheep, a rapid (within 2–6 h) decrease in GnRH pulse frequency was observed with progesterone administration (Bergfeld et al., 1996; Skinner et al., 1998). While two studies in women suggested that LH pulse frequency can be suppressed within 8–14 h of progesterone administration (Minakami et al., 1984; Permezel et al., 1989), two others suggested that progesterone administration does not suppress LH pulse frequency within 12 h (Hutchens et al., 2016; McCartney et al., 2007). In addition, the GnRH pulse generator is relatively resistant to progesterone negative feedback in women with polycystic ovary syndrome (PCOS) (Daniels & Berga, 1997; Pastor et al., 1998), and the time course of progesterone‐related LH pulse frequency lowering in PCOS is similarly unclear.

In addition to the negative feedback effects of progesterone on LH pulse frequency, progesterone can acutely augment gonadotropin release. Prior studies disclosed a rapid (within 4 h) and marked augmentation of LH and FSH secretion after oral progesterone administration in normally cycling women pretreated with estradiol (Hutchens et al., 2016; McCartney et al., 2007), similar to previous reports (Chang & Jaffe, 1978; Nippoldt et al., 1987). Although the positive feedback phenomenon accounting for the midcycle gonadotropin surge primarily reflects estradiol positive feedback, a pre‐ovulatory increase in circulating progesterone may augment estradiol positive feedback at midcycle (Chang & Jaffe, 1978; Liu & Yen, 1983). Indeed, some studies suggest that progesterone positive feedback is important for normal surge characteristics, including the FSH surge (Chang & Jaffe, 1978; Liu & Yen, 1983; March et al., 1979; Taylor et al., 1995). Although women with PCOS demonstrate impaired progesterone negative feedback on LH pulse frequency, it remains unclear whether such women may also demonstrate impaired progesterone positive feedback.

The purpose of this study was to test the primary hypothesis that exogenous progesterone, given in the early morning, acutely (within 12 h) suppresses waking LH pulse frequency in estradiol‐pretreated normally cycling women, but to a lesser degree in estradiol‐pretreated women with PCOS (i.e., that acute progesterone negative feedback on waking LH pulse frequency is impaired in PCOS). An a priori secondary hypothesis was that exogenous progesterone acutely increases LH and FSH concentrations more so in normally cycling women compared to women with PCOS (i.e., that acute progesterone positive feedback on gonadotropin release is impaired in PCOS). Some data from this study was previously reported: in 12 normally cycling women (the control group), progesterone administration did not acutely reduce waking LH pulse frequency, although progesterone acutely and markedly increased LH and FSH concentrations (Hutchens et al., 2016). Herein findings in the PCOS group are reported, in addition to comparisons between PCOS and controls.

## 2 MATERIALS AND METHODS

All study procedures, which were in accordance with the ethical standards of Helsinki Declaration of 1975, as revised in 2008, were approved by the Institutional Review Board for Health Sciences Research at the University of Virginia (UVA). The study was registered with ClinicalTrials.gov (NCT00594217).

### 2.1 Subjects

Twelve normally cycling controls and 12 women with PCOS completed the study. The study was initiated in both groups contemporaneously. However, subject accruement was achieved more rapidly for normally cycling controls. All 12 controls completed study from 2009 to 2012. Seven women with PCOS completed study from 2010 to 2013, and five completed study between 2014 and 2020. Findings in normally cycling controls were previously reported (Hutchens et al., 2016).

As previously described, controls had regular menstrual cycles and were without evidence of hyperandrogenism. Subjects were considered to have PCOS if they had evidence of clinical and/or biochemical hyperandrogenism plus oligo‐/amenorrhea in the absence of other identifiable causes. Subjects had not taken any medications known to affect the reproductive axis for 90 days prior to and during the study. A detailed description of inclusion and exclusion criteria used for the study are available in supplemental materials. Baseline characteristics of all participants are summarized in Table 1.

**TABLE 1:** Baseline characteristics

Variable	NC (*n* = 12) (median [IQR])	PCOS (*n* = 12) (median [IQR])	*p* value
Age (years)	19 [18.8–20.0]	25.5 [20.8–27.3]	0.029
Body mass index (kg/m^2^)	21.8 [20.4–23.0]	29.9 [25.5–35.9]	0.006
Body fat percentage (%)	23.3 [21.0–27.9]	42.4 [31.0–46.2]	0.006
Waist‐to‐hip ratio	0.76 [0.72–0.79]	0.81 [0.77–0.84]	0.118
Total testosterone (ng/dL)*	18.0 [14.6–23.8]	42.4 [35.2–69.7]	<0.001
Sex hormone‐binding globulin (nmol/L)	39.0 [30.6–52.3]	31.2 [19.6–40.9]	0.149
Free testosterone (pg/mL)	10.4 [2.6–13.4]	34.6 [19.5–41.5]	<0.001
LH (IU/L)*	7.7 [4.9–10.1]	12.3 [9.7–19.8]	0.010
FSH (IU/L)*	4.2 [3.3–5.1]	4.1 [3.1– 5.7]	0.729
Fasting insulin (μIU/mL)	2.4 [2.0–5.1]	9.9 [6.1–15.1]	0.001
Fasting glucose (mg/dL)	87.0 [82.0–87.0]	91.5 [87.8–95.0]	0.007
Hemoglobin A1c (%)	5.2 [5.1–5.4]	5.2 [4.9–5.5]	0.954

Note

Group comparisons were performed via Wilcoxon Rank Sum tests. To convert units to SI units: total testosterone (ng/dL) × 0.0347 (nmol/L); free testosterone (pg/mL) × 3.467 (pmol/L); insulin (μIU/mL) × 7.175 (pmol/L); glucose (mg/dL) × 0.0555 (nmol/L).

Abbreviations: IQR, interquartile range; NC, normally cycling control; PCOS, polycystic ovary syndrome.

### 2.2 Study procedures

After informed consent was obtained, volunteers underwent a screening history, physical examination, and bloodwork to ensure they met all study eligibility criteria. All participants had normal screening tests except for abnormalities that were expected in the PCOS group (e.g., hyperandrogenemia).

**FIGURE 1:**
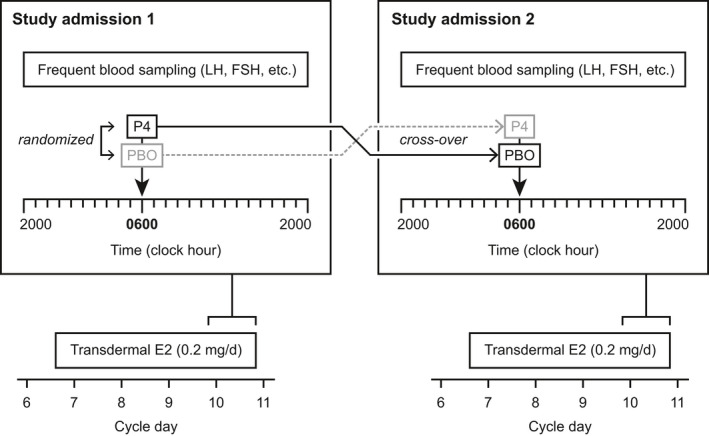
Schematic of study protocol. With regard to the order of progesterone and placebo administration, the solid line illustrates initial randomization to progesterone, while the dotted line illustrates initial randomization to placebo

This was a randomized, double‐blinded, placebo‐controlled, crossover study. Each subject underwent two frequent sampling studies to characterize LH and FSH secretion (Figure 1). Each subject was randomized to receive either progesterone or placebo during her first 24‐h admission to the Clinical Research Unit (CRU). During a second 24‐h CRU admission, which occurred in a subsequent menstrual cycle, subjects received the intervention (placebo or progesterone) that they did not receive during the first admission. (In all cases, the second admission occurred at least 28 days after the first admission.) Therefore, when assessing the acute impact of progesterone versus placebo on gonadotropin release, each subject served as her own control.

Beta‐hCG and progesterone levels were checked 4–5 days before scheduled CRU admissions to rule out pregnancy and, in women with PCOS, to help ensure the study would not be performed during a luteal phase. Starting 3 days before each admission, subjects were pretreated with estradiol patches (delivering a total dose of 0.2 mg/day) placed on the abdomen and changed after 2 days. Estradiol was administered to standardize hypothalamic progesterone receptors. Normally cycling women started estradiol on cycle days 4–8 inclusive; subjects with PCOS started estradiol no earlier than cycle day 4. Subjects continued estradiol throughout each overnight admission.

On day 3 of estradiol administration—cycle days 7–11 inclusive in controls—subjects were admitted to CRU at 18:00 h for a 24‐h frequent blood sampling study. Beginning at 20:00 h, blood was obtained through an indwelling intravenous forearm catheter over a 24‐h period as follows: LH every 10 min; progesterone every 30 min; FSH, estradiol, and testosterone every 120 min. At 06:00 h, after obtaining additional blood for SHBG, fasting insulin, and fasting glucose, either oral micronized progesterone (100 mg) or placebo suspension was administered. At the completion of blood sampling at 20:00 h, estradiol patches were removed and subjects were discharged. In a subsequent cycle, subjects underwent another overnight study identical to the first except that oral progesterone was exchanged for placebo or vice versa in keeping with the crossover design.

During each admission, subjects fasted from 22:00 to 06:00 h; they were otherwise given standard meals at standard times during the admission. In addition, subjects were encouraged to sleep from 22:00 to 06:00 h, and they were not allowed to sleep before 22:00 h or after 06:00 h. Sleep was evaluated via Motionlogger Basic‐L wrist actigraphy. At discharge from both admissions, subjects were provided and advised to take oral iron supplementation (325 mg twice daily for 30 days) to help replenish iron stores.

### 2.3 Hormonal measurements

The Ligand Assay and Analysis Core of the Center for Research in Reproduction (CRR) performed all hormonal assays as previously described (Hutchens et al., 2016). Detailed assay characteristics were described previously (Kim et al., 2018). In brief, LH and FSH were measured by chemiluminescence (sensitivities 0.1 and 0.1 IU/L; intraassay coefficient of variation [CVs] 3.3 and 3.2%; interassay CVs 5.8 and 4.9%; Siemens Healthcare Diagnostics, Los Angeles, CA). Prior to January 2015, sex steroids were measured by radioimmunoassay (RIA; Diagnostic Products, DPC). In 2014, the manufacturer discontinued most steroid RIA kits used by the CRR. Therefore, starting January 2015, the total testosterone, estradiol, and progesterone were measured by other immunoassays. Each replacement method was evaluated according to the Endocrine Society Sex Steroid Assays Reporting Task Force guidelines (Wierman et al., 2014). The replacement methods were correlated to DPC RIA, and the RIA‐equivalent values were used for analysis and presented here. Importantly, all assay methods were identical between admissions for each study participant.

### 2.4 Data analysis

To account for unequal LH measurement error across the physiologic range of LH concentrations observed during study admissions, a computerized data reduction protocol (*StdCurve*) was used to generate a variance model for LH measurement error for each admission, as described previously (Hutchens et al., 2016). Pulsatile LH secretion was then assessed for each 10‐h time block of interest (see below) using *AutoDecon*, an automated (non‐subjective) multiparameter deconvolution program, as described previously (Hutchens et al., 2016). *AutoDecon* has 96% sensitivity to detect LH pulses with a low (6%) false positive rate (Johnson et al., 2008). In addition to determining the temporal location of each significant LH pulse, *AutoDecon* provides estimates for mean LH pulse mass, area under the LH concentration curve (AUC), basal (non‐pulsatile) LH secretion, and LH half‐life in the circulation. LH pulse mass—a correlate of LH pulse amplitude—is an estimate of the amount of LH secreted by the pituitary during each pulse.

The primary outcome variable was LH pulse frequency. Using *AutoDecon*‐identified LH pulse locations, average interpulse interval (IPI) was defined for specified time blocks, as previously described (McCartney et al., 2007). LH pulse frequency (pulses/hour) over the specified time block was calculated as 60 divided by average IPI. Secondary outcome variables included mean LH, mean FSH, LH AUC, FSH AUC, mean LH pulse mass, pulsatile LH secretion, basal LH secretion, and LH half‐life. Pulsatile LH secretion was calculated for each time block as average LH pulse mass multiplied by the number of LH pulses. Notably, predefined pulse exclusion criteria (as previously described (Hutchens et al., 2016)) were not applied given that doing so would lead to analytical errors with regard to *AutoDecon*‐derived LH AUC and mean LH pulse mass. The AUC of FSH concentration during each time block was calculated using the trapezoidal rule.

The primary a priori hypotheses were as follows: in normally cycling women, the pre‐ to post‐progesterone reduction in 10‐h LH pulse frequency (20:00–06:00 vs. 10:00–20:00 h) would be greater than the pre‐ to post‐placebo reduction in 10‐h LH pulse frequency, and the progesterone‐related reduction in 10‐h LH pulse frequency (a negative feedback effect) would be less prominent in women with PCOS compared to normally cycling controls. A priori secondary hypotheses were that progesterone‐related increases in LH and FSH release (positive feedback effects) would be less prominent in women with PCOS compared to normally cycling controls.

### 2.5 Statistical analysis

Based on previously obtained data regarding within‐subject variability in LH pulse frequency differences between progesterone and placebo treatments (McCartney et al., 2007), it was determined that 12 normally cycling women without hyperandrogenism would need to complete the current study to detect a 1.0 mean change in 10‐h LH pulse frequency (16.7‐min mean change in 10‐hour LH interpulse interval) between the progesterone and placebo conditions, assuming 80% statistical power and 0.05 type I error rate. Based on the assumption that the PCOS group would not demonstrate a short‐term progesterone‐related change in LH pulse frequency, and that within‐subject variability in PCOS group would be similar to that in controls, we reasoned that 12 subjects in the PCOS group would provide a minimum detectable absolute progesterone‐related interpulse interval change of 16.7 min between the PCOS and control groups with at least 80% statistical power. Sixteen normally cycling women and 13 women with PCOS initiated formal study procedures. However, for personal reasons, 3 women in the control group and one woman in the PCOS group dropped out of the study after completing only one admission. In addition, data from one woman in the control group was excluded from analysis because her average progesterone level before progesterone administration was 4.8 ng/mL (15.3 nmol/L), indicating that the frequent‐sampling study was unintentionally performed during the luteal phase. Thus, data for 12 women in each study group were formally analyzed.

Statistical analyses were conducted using a 2 × 2 crossover design ANOVA paradigm to compare the baseline (pre‐intervention) outcome measure (e.g., pre‐intervention LH pulse frequency) between the placebo and progesterone admissions for each study group, and to compare the within‐group (PCOS or non‐PCOS) pre‐ to post‐intervention changes in the outcome measure between the placebo and progesterone admissions (e.g., pre‐ to post‐progesterone change in LH pulse frequency vs. the pre‐ to post‐placebo change in LH pulse frequency). A 2 × 2 crossover design ANOVA paradigm was also used to compare progesterone‐attributable changes (i.e., pre‐ to post‐progesterone changes vs. pre‐ to post‐placebo changes) in the outcome measure between the PCOS and control groups.

All outcome measures were analyzed on a natural logarithmic scale to satisfy the assumptions of ANOVA (parametric) testing, and all hypothesis testing was directed at comparing the geometric mean of the measurement distribution. The geometric mean (GM) is a central location parameter of the measurement distribution; it is analogous to arithmetic mean of the measurement distribution, but is less sensitive than the arithmetic mean to be influenced by positively skewed data when the outcome measure is restricted to positive values.

With regard to hypothesis testing for the pre‐intervention outcome measure comparisons, an *F*‐test was used to test the null hypothesis that the GM of the measurement distribution is the same for the placebo and progesterone admissions. For the within‐group comparisons (PCOS or non‐PCOS) of the pre‐ to post‐intervention (placebo or progesterone) change in outcome measure, an *F*‐test was used to test the null hypothesis that the ratio of GMs (i.e., either post‐placebo GM:pre‐placebo GM or post‐progesterone GM:pre‐progesterone GM) is equal to 1. For the within‐group (PCOS or non‐PCOS) between‐admission comparisons (placebo vs. progesterone), an *F*‐test was used to test the null hypothesis that the ratio of GMs (i.e. post‐intervention GM:pre‐intervention GM) is the same irrespective of the intervention. Finally, for the between‐group (PCOS vs. non‐PCOS) between‐admission comparisons (i.e., placebo vs. progesterone admissions), an *F*‐test was used to test the null hypothesis that the between‐admission ratio of intra‐admission GM ratios (i.e. post‐intervention:pre‐intervention) is the same irrespective of the study group (PCOS vs. non‐PCOS). Due to the multiple hypothesis testing strategy, all of the aforementioned within‐group and between‐group hypothesis tests were analytically assessed for statistical significance based on a multiple comparison Bonferroni‐corrected *p* ≤ 0.05 criterion.

As sensitivity analyses, two additional sets of post hoc analyses were performed. First, given that group differences in achieved progesterone and estradiol concentrations could have represented confounders in the primary (a priori) analyses, the above analyses were repeated using a 2 × 2 crossover ANCOVA paradigm to adjust for differences in achieved progesterone and estradiol levels. Second, one normally cycling subject had a marked discordance of mean LH and LH pulse amplitude responses to progesterone/placebo compared to the other participants, as previously reported (Hutchens et al., 2016). To address the possibility that samples for her progesterone and placebo admissions had been mislabeled, all of the aforementioned analyses were repeated after excluding this subject.

Unless otherwise specified, uncorrected *p* values are reported herein. Both uncorrected and Bonferroni‐corrected *p* values are routinely reported whenever they fall on different sides of the 0.05 threshold. More detailed results of statistical analyses are available in supplemental materials.

## 3 RESULTS

The progesterone admission occurred first in 2 of 12 controls and in 8 of 12 women with PCOS. The progesterone admission occurred on cycle day 10 (9–11) (median [interquartile range, IQR]) for normally cycling controls, and cycle day 45.0 (12.5–61.0) for women with PCOS. The placebo admissions occurred on cycle days 10.0 (9.0–11.0) and 30.0 (12.8–59.3) for the control and PCOS groups, respectively. As assessed with wrist actigraphy, respective sleep durations (median [IQR]) for progesterone and placebo admissions were as follows: 7.8 (7.4–7.9) h and 7.2 (6.6–7.7) h in controls; 7.8 (7.1–7.8) h and 7.7 (7.3–7.8) h for PCOS. Respective sleep efficiencies for progesterone and placebo admissions were as follows: 93 (90–94) and 97 (94–99) percent in controls; 89 (87–99) and 92 (87–95) percent in PCOS.

### 3.1 Sex steroids

**FIGURE 2:**
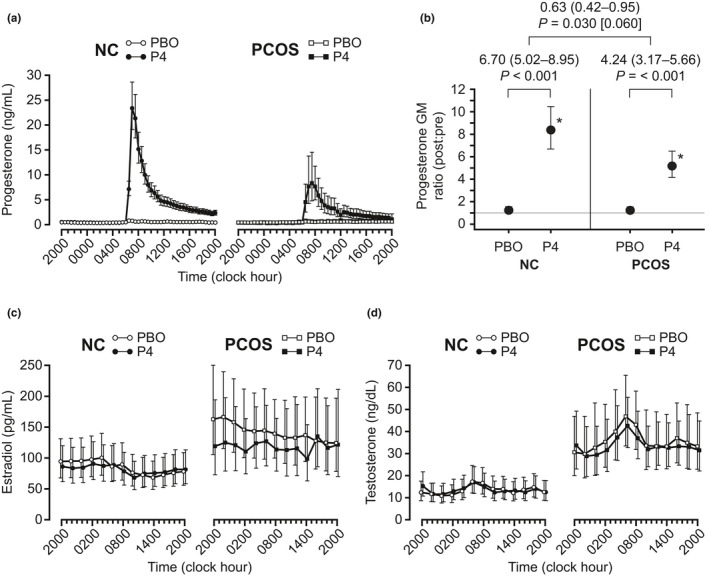
Serum sex steroid concentrations in normally cycling control (NC) and polycystic ovary syndrome (PCOS) groups. (a) *(progesterone)*, (c) *(estradiol)*, *and* (d) *(testosterone)*: Data from the control group are shown on the left (open circles indicate placebo [PBO] admissions; closed circles indicate progesterone [P4] admissions). Data from the PCOS group are shown on the right (open squares indicate PBO admissions; closed squares indicate P4 admissions). Each data point represents the group geometric mean (GM) for that time point, with vertical lines indicating 95% confidence intervals. (b) Post‐intervention relative to pre‐intervention progesterone concentrations for the PBO and P4 admissions, expressed as GM ratios. Vertical lines identify the 95% confidence interval for the GM ratio; the gray horizontal line identifies the GM ratio of equality (i.e., where GM ratio = 1); and asterisks denote statistically significant changes (*p* < 0.05, pre‐ vs. post‐intervention). For each group, between‐admission comparisons of pre‐ versus post‐intervention changes (i.e., pre‐ vs. post‐placebo change vs. pre‐ vs. post‐progesterone change)—reflecting changes attributable to progesterone administration—are expressed as a ratio of GM ratios with accompanying 95% confidence intervals. Accompanying *p* values are for the null hypothesis test that the GM ratio is the same for the PBO and P4 admissions. Finally, between‐group comparisons of such GM ratios—reflecting changes attributable to progesterone administration in the control group vs. changes attributable to progesterone administration in the PCOS group—are expressed as a ratio of GM ratios with accompanying 95% confidence intervals. For this between‐group comparison, the unadjusted *p* < 0.05 but the Bonferroni‐adjusted *p* value (in brackets) was >0.05. To convert metric units to SI units: progesterone × 3.18 (nmol/L); estradiol × 3.67 (pmol/L); testosterone × 0.0347 (nmol/L)

Progesterone, estradiol, and total testosterone concentrations during the admissions are summarized in Table 2, and the respective hormone time series are presented graphically in Figure 2. Pre‐intervention 10‐h (20:00–06:00 h) progesterone concentrations were similar between placebo and progesterone admissions for both normal controls and PCOS (*p* > 0.5 for both groups). Ten‐hour progesterone concentrations increased markedly with progesterone administration in both groups (8.4‐fold increase in GM [95% CI, 6.7–10.5] in controls; 5.2‐fold increase in GM [95% CI, 4.2–6.5] in PCOS; *p* < 0.001 for both groups). Ten‐hour progesterone concentrations increased slightly with placebo, although such changes were not statistically significant in the control group (25% increase in GM [95% CI, 0–56%]; *p* = 0.052) or in the PCOS group (23% increase in GM [95% CI, −2–53%]; *p* = 0.074). As expected, the change in 10‐h progesterone concentrations associated with progesterone administration exceeded the change associated with placebo administration in both groups (6.7‐fold higher in controls; 4.2‐fold higher in PCOS; *p* < 0.001 for both; Figure 2B). Notably, the change in 10‐hour progesterone concentrations attributable to progesterone administration appeared to be less pronounced in PCOS compared to controls (ratio of ratios 0.63 [95% CI, 0.42–0.95]; *p* = 0.030), although this was not statistically significant after Bonferroni correction (*p* = 0.060).

**TABLE 2:** Sex steroid concentrations

Variable	Admission/intervention	Assessment time block (clock hours)	NC (*n* = 12) (median [IQR])	PCOS (*n* = 12) (median [IQR])
Progesterone (ng/ml)	PBO	20:00–06:00 (pre‐PBO)	0.4 [0.3–0.6]	0.5 [0.2–1.8]
		10:00–20:00 (post‐PBO)	0.6 [0.4–0.8]	0.7 [0.2–2.8]
	P4	20:00–06:00 (pre‐P4)	0.5 [0.2–0.8]	0.4 [0.3–0.8]
		10:00–20:00 (post‐P4)	3.7 [2.4–7.1]	2.2 [1.1–6.0]
Estradiol (pg/ml)	PBO	20:00–20:00	81 [38–162]	148 [48–275]
	P4	20:00–20:00	76 [28–224]	113 [35–261]
Total testosterone (ng/dl)	PBO	20:00–20:00	14.2 [5.1–32.2]	32.9 [18.3–76.5]
	P4	20:00–20:00	14.7 [6.1–28.0]	31.4 [19.3–77.7]

Note

Summary statistics (median and interquartile range) are shown for average progesterone concentrations during the 10‐h pre‐intervention (20:00–06:00 h) and 10‐hour post‐intervention (10:00–20:00 h) time blocks. Summary statistics are also shown for average testosterone and estradiol concentrations for the entire admission (20:00–20:00 h). More detailed summary statistics are included in supplemental materials. The number of subjects is 12 for both groups. To convert metric units to SI units: progesterone × 3.18 (nmol/L); estradiol × 3.67 (pmol/L); testosterone × 0.0347 (nmol/L).

Abbreviations: IQR, interquartile range; NC, normally cycling control; P4, progesterone; PBO, placebo; PCOS, polycystic ovary syndrome.

In both groups, mean estradiol and total testosterone levels (Figure 2) were similar between the progesterone and placebo admissions. Achieved estradiol levels were not significantly different between controls and PCOS (*p* = 0.065 and 0.192 for the placebo and progesterone admissions, respectively). As expected, total testosterone was higher in the PCOS groups (*p* < 0.001).

### 3.2 LH pulse frequency

**FIGURE 3:**
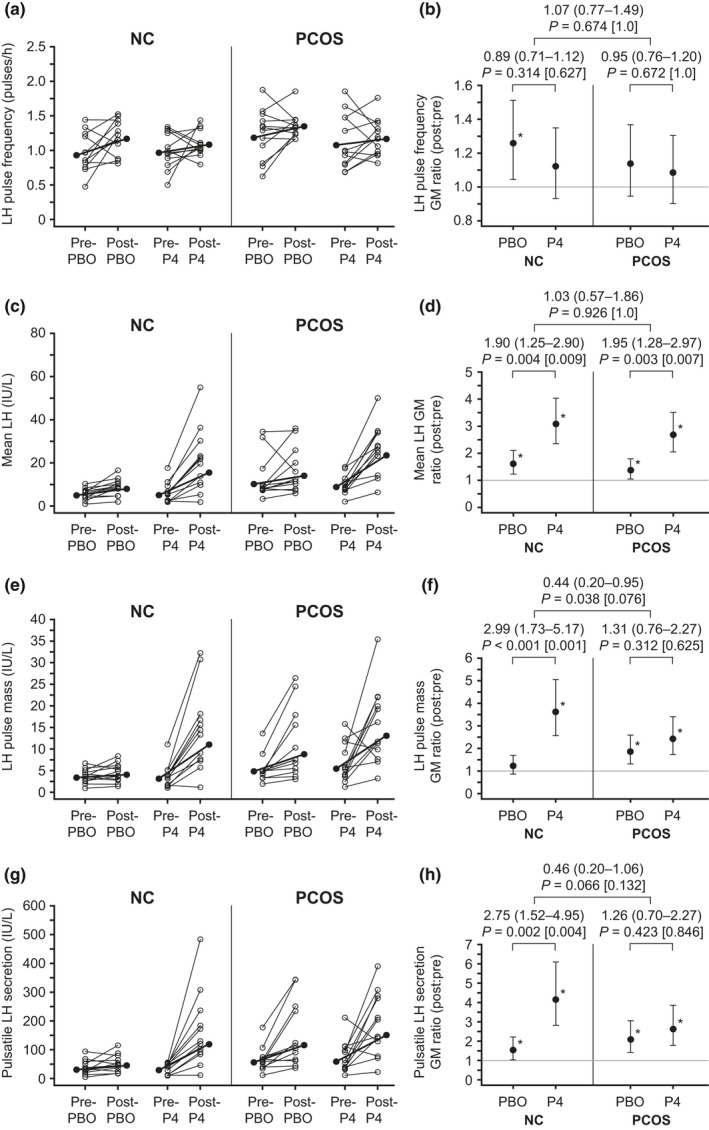
LH secretory characteristics in normally cycling control (NC) and polycystic ovary syndrome (PCOS) groups: LH pulse frequency (a and b), mean LH (c and d), LH pulse mass (e and f), and pulsatile LH secretion (g and h). (a, c, e, g): Individual subject changes for control (left) and PCOS (right) groups. For each group, open circles connected by a thin line indicate individual subject changes (pre‐intervention to post‐intervention) for placebo (PBO; left) and progesterone (P4; right) admissions. Solid circles connected by a thick line indicate group geometric mean (GM) changes (pre‐ to post‐intervention). (b, d, f, h): Post‐intervention relative to pre‐intervention LH characteristics for the PBO and P4 admissions, expressed as GM ratios. Vertical lines identify the 95% confidence interval for the GM ratio; the gray horizontal line identifies the GM ratio of equality (i.e., where GM ratio = 1); and asterisks denote statistically significant changes (*p* < 0.05, pre‐ vs. post‐intervention). For each group, between‐admission comparisons of pre‐ versus post‐intervention changes (i.e., the pre‐ vs. post‐placebo change vs. the pre‐ vs. post‐progesterone change)—reflecting changes attributable to progesterone administration—are expressed as a ratio of GM ratios with accompanying 95% confidence intervals. Unadjusted *p* values (and Bonferroni‐adjusted *p* values in brackets) are for the null hypothesis test that changes accompanying PBO administration are the same as changes accompanying P4 administration. Between‐group comparisons of such GM ratios (i.e., changes attributable to progesterone administration in the control group vs. changes attributable to progesterone administration in the PCOS group) are also expressed as a ratio of GM ratios with accompanying 95% confidence intervals. Unadjusted *p* values (and Bonferroni‐adjusted *p* values in brackets) are for the null hypothesis test that changes attributable to progesterone in the control group are the same as changes attributable to progesterone in the PCOS group

Simple summary statistics for LH pulse frequency are shown in Table 3 (more detail is provided in supplemental materials), and intervention‐related changes are graphically represented in Figure 3. Pre‐intervention LH pulse frequency was similar between placebo and progesterone admissions (*p* > 0.3) in both groups. In controls, 10‐h GM LH pulse frequency increased by 26% (95% CI, 4–52%; *p* = 0.017) and 12% (95% CI, −7–35%; *p* = 0.221) with placebo and progesterone administration, respectively, with no significant difference between placebo and progesterone (ratio of GM ratios 0.89 [95% CI 0.71–1.12]; *p* = 0.314). In women with PCOS, 10‐h GM LH pulse frequency increased by 14% (95% CI, −6–37%; *p* = 0.168) and 8% (95% CI −10–31%; *p* = 0.383) with placebo and progesterone administration, respectively, with no significant difference between placebo and progesterone (ratio of GM ratios 0.95 [95% CI 0.76–1.20]; *p* = 0.672). The changes in 10‐h LH pulse frequency attributable to progesterone were similar between the control and PCOS groups (ratio of ratios 1.07 [95% CI, 0.77–1.49]; *p* = 0.674). Overall, these results suggest that exogenous progesterone does not acutely reduce LH pulse frequency in normally cycling controls or in women with PCOS.

**TABLE 3:** Selected gonadotropin characteristics

Variable	Admission/intervention	Assessment time block (clock hours)	NC (*n* = 12) (median [IQR])	PCOS (*n* = 12) (median [IQR])
LH pulse frequency (pulses/hour)	PBO	20:00–06:00 (pre‐PBO)	0.91 [0.81–1.44]	1.31 [1.01–1.88]
		10:00–20:00 (post‐PBO)	1.19 [1.04–1.52]	1.33 [1.24–1.85]
	P4	20:00–06:00 (pre‐P4)	0.98 [0.86–1.34]	0.99 [0.80–1.85]
		10:00–20:00 (post‐P4)	1.05 [1.01–1.44]	1.18 [0.97–1.76]
Mean LH (IU/L)	PBO	20:00–06:00 (pre‐PBO)	6.4 [3.9–10.3]	8.2 [7.8–34.4]
		10:00–20:00 (post‐PBO)	8.8 [7.1–16.5]	12.0 [9.6–36.0]
	P4	20:00–06:00 (pre‐P4)	5.6 [2.5–17.6]	8.6 [7.6–18.1]
		10:00–20:00 (post‐P4)	20.7 [10.6–54.9]	26.6 [20.9–50.0]
LH AUC	PBO	20:00–06:00 (pre‐PBO)	3756 [2330–6171]	4760 [4346–20264]
		10:00–20:00 (post‐PBO)	5166 [2842–9847]	7129 [5997–23131]
	P4	20:00–06:00 (pre‐P4)	3330 [1498–10465]	5654 [4704–18507]
		10:00–20:00 (post‐P4)	12214 [6514–32873]	14992 [9272–29775]
LH pulse mass (IU/L)	PBO	20:00–06:00 (pre‐PBO)	3.6 [2.5–6.7]	4.85 [4.0–13.6]
		10:00–20:00 (post‐PBO)	4.3 [2.9–8.2]	7.51 [5.3–26.4]
	P4	20:00–06:00 (pre‐P4)	3.6 [1.7–11.1]	5.47 [3.8–15.8]
		10:00–20:00 (post‐P4)	13.9 [7.5–32.2]	13.96 [9.3–35.4]
Pulsatile LH secretion (IU/L)	PBO	20:00–06:00 (pre‐PBO)	31.0 [23.8–93.5]	60.6 [40.3–176.7]
		10:00–20:00 (post‐PBO)	46.9 [34.2–114.9]	108.7 [64.1–343.5]
	P4	20:00–06:00 (pre‐P4)	43.3 [11.9–55.3]	58.4 [35.1–210.8]
		10:00–20:00 (post‐P4)	124.7 [89.1–483.5]	174.8 [115.4–389.4]
Mean FSH (IU/L)	PBO	20:00–06:00 (pre‐PBO)	3.5 [3.1–5.4]	4.5 [3.0–6.3]
		10:00–20:00 (post‐PBO)	4.5 [3.8–7.0]	3.9 [3.3–9.0]
	P4	20:00–06:00 (pre‐P4)	4.4 [2.5–5.9]	4.2 [3.0–6.4]
		10:00–20:00 (post‐P4)	8.0 [5.63–14.6]	6.3 [4.4–9.1]
FSH AUC	PBO	20:00–06:00 (pre‐PBO)	34.5 [30.5–53.0]	43.1 [29.4–57.8]
		10:00–20:00 (post‐PBO)	45.0 [36.7–70.7]	38.1 [27.5–89.9]
	P4	20:00–06:00 (pre‐P4)	42.4 [24.8–59.0]	41.7 [29.5–63.2]
		10:00–20:00 (post‐P4)	79.8 [57.6–151.2]	65.7 [45.9–93.5]

Note

Summary statistics (median and interquartile range) are shown for selected gonadotropin characteristics—LH pulse frequency, mean LH, LH area under the curve, LH pulse mass, pulsatile LH secretion, mean FSH, and FSH AUC—during the 10‐h pre‐intervention (20:00–06:00 h) and 10‐h post‐intervention (10:00–20:00 h) time blocks. Additional data (basal LH secretion and LH half‐life) and more detailed summary statistics are included in supplemental materials. The number of subjects is 12 for both groups.

Abbreviations: IQR, interquartile range; NC, normally cycling control; P4, progesterone; PBO, placebo; PCOS, polycystic ovary syndrome.

### 3.3 Mean LH and LH AUC

**FIGURE 4:**
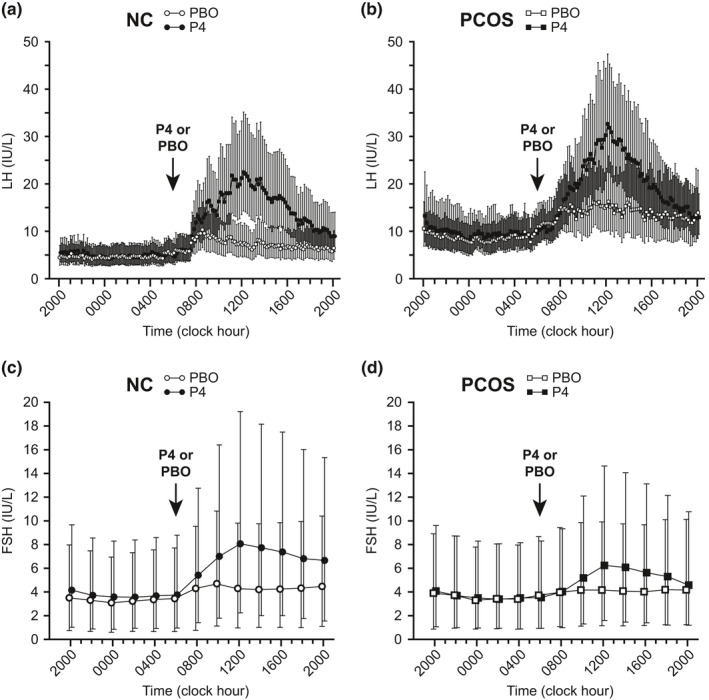
LH and FSH time series for normally cycling control (NC) and polycystic ovary syndrome (PCOS) groups. Data from the control group (a, LH; c, FSH) are shown as open (placebo [PBO] admission) and closed (progesterone [P4] admission) circles. Data from the PCOS group (b, LH; d, FSH) are shown as open (PBO admission) and closed (P4 admission) squares. Each data point represents the group geometric mean for that time point, with vertical lines indicating 95% confidence intervals. The time points at which either PBO or P4 was administered is indicated by the arrows

Pre‐intervention mean LH was similar between placebo and progesterone admissions in both groups (*p* > 0.4 for both) (Table 3; Figures 3 and 4). In controls, 10‐h GM mean LH increased 1.61‐fold (95% CI, 1.23–2.11; *p* = 0.001) and 3.07‐fold (95% CI, 2.34–4.02; *p* < 0.001) with placebo and progesterone administration, respectively, with a significantly greater increase with progesterone administration (ratio of GM ratios 1.90 [95% CI, 1.25–2.90]; *p* = 0.004). In women with PCOS, 10‐h GM mean LH increased by 1.37‐fold (95% CI, 1.05–1.80; *p* = 0.022) and 2.68‐fold (95% CI, 2.05–3.51; *p* < 0.001) with placebo and progesterone administration, respectively, with a significantly greater increase with progesterone administration (ratio of ratios 1.95 [95% CI, 1.28–2.97]; *p* = 0.003). The changes attributable to progesterone were similar between the control and PCOS groups (*p* = 0.926).

LH area under the curve (AUC) results (Table 3) were generally similar to those for mean LH. Controls exhibited a significantly greater increase in LH AUC with progesterone versus placebo administration (ratio of GM ratios 2.22 [95% CI, 1.36–3.61]; *p* = 0.003), but women with PCOS did not (ratio of GM ratios 1.41 [95% CI, 0.86–2.30]; *p* = 0.160). However, changes in LH AUC attributable to progesterone were not demonstrably different between the control and PCOS groups (*p* = 0.187).

Taken together, these results confirm that progesterone acutely augments mean LH and LH AUC in E2‐pretreated women, but they do not substantively support the hypothesis that acute progesterone augmentation of mean LH or LH AUC is impaired in women with PCOS.

### 3.4 LH pulse mass and pulsatile LH secretion

Pre‐intervention LH pulse mass was similar between placebo and progesterone admissions for both groups (*p* > 0.4 for both) (Table 3, Figure 3). In controls, 10‐h GM LH pulse mass increased by 20% (95% CI, −14–69%; *p* = 0.277) and 3.60‐fold (95% CI, 2.56–5.05; *p* < 0.001) with placebo and progesterone administration, respectively, with a significantly greater increase with progesterone (ratio of GM ratios 2.99 [95% CI 1.73–5.17]; *p* < 0.001). In women with PCOS, 10‐h GM LH pulse mass increased by 84% with placebo (95% CI, 31–159%; *p* = 0.001) and 2.42‐fold with progesterone (95% CI, 1.72–3.40; *p* < 0.001), with no significant difference between placebo and progesterone (ratio of GM ratios 1.31 [95% CI, 0.76–2.27]; *p* = 0.312). The changes in 10‐h LH pulse mass attributable to progesterone were less prominent in the PCOS group compared to controls (ratio of ratios 0.44 [95% CI, 0.20–0.95]; *p* = 0.038), although this was not significant after Bonferroni correction (*p* = 0.076).

Pre‐intervention pulsatile LH secretion was similar between placebo and progesterone admissions in controls and in women with PCOS (*p* > 0.7 for both) (Table 3; Figure 3). In controls, 10‐h GM LH pulsatile secretion increased 51% (95% CI, 3–122%; *p* = 0.038) and 4.14‐fold (95% CI, 2.82–6.09; *p* < 0.001) with placebo and progesterone administration, respectively, with a significantly greater increase with progesterone administration (ratio of GM ratios 2.75 [95% CI, 1.52–4.95]; *p* = 0.002). In women with PCOS, 10‐h GM LH pulsatile secretion increased 2.08‐fold (95% CI, 1.41–3.05; *p* < 0.001) and 2.62‐fold (95% CI, 1.78–3.85; *p* < 0.001) with placebo and progesterone administration, respectively, with no significant difference between the two (GM ratio 1.26 [95% CI, 0.70–2.27]; *p* = 0.423). The changes in 10‐h pulsatile LH secretion attributable to progesterone appeared to be less pronounced in women with PCOS compared to controls, although this was not statistically significant (ratio of ratios 0.46 [95% CI, 0.20–1.06]; *p* = 0.066).

Overall, these results suggest that progesterone acutely augments LH pulse mass and LH pulsatile secretion in E2‐pretreated women. Although not conclusive, these results also suggest the possibility that acute progesterone augmentation of LH pulse mass and pulsatile LH secretion may be impaired in PCOS.

### 3.5 Basal LH secretion and LH half‐life

Pre‐intervention LH basal secretion and LH half‐life were similar between placebo and progesterone admissions in the control and PCOS groups. Neither basal LH secretion nor LH half‐life changed substantially with placebo or progesterone administration; no significant differences in basal LH secretion or LH half‐life were observed between the placebo and progesterone conditions in either group (supplemental materials); and no progesterone‐related differences were observed between the control and PCOS groups for 10‐h basal LH secretion (*p* = 0.735) or for 10‐h LH half‐life (*p* = 0.261). Overall, these results suggest that exogenous progesterone does not acutely affect basal LH secretion or LH half‐life in normally cycling controls or in women with PCOS.

### 3.6 Mean FSH and FSH AUC

**FIGURE 5:**
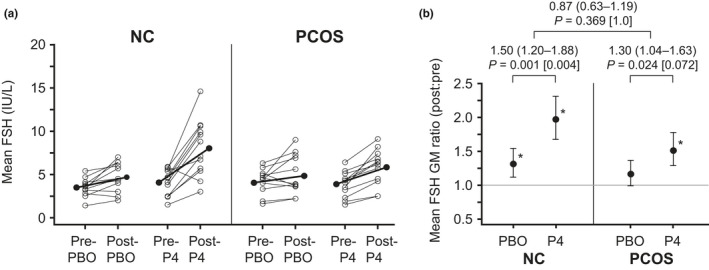
FSH concentrations in normally cycling control (NC) and polycystic ovary syndrome (PCOS) groups. (a) Individual subject changes for control (left) and PCOS (right) groups. For each group, open circles connected by a thin line indicate individual subject changes (pre‐intervention to post‐intervention) for placebo (PBO; left) and progesterone (P4; right) admissions. Solid circles connected by a thick line indicate group geometric mean (GM) changes (pre‐ to post‐intervention). (b) Post‐intervention relative to pre‐intervention FSH for the PBO and P4 admissions, expressed as GM ratios. Vertical lines identify the 95% confidence interval for the GM ratio; the gray horizontal line identifies the GM ratio of equality (i.e., where GM ratio = 1); and asterisks denote statistically significant changes (*p* < 0.05, pre‐ vs. post‐intervention). For each group, between‐admission comparisons of pre‐ versus post‐intervention changes (i.e., the pre‐ vs. post‐placebo change vs. the pre‐ vs. post‐progesterone change)—reflecting changes attributable to progesterone administration—are expressed as a ratio of GM ratios with accompanying 95% confidence intervals. Unadjusted *p* values (and Bonferroni‐adjusted *p* values in brackets) are for the null hypothesis test that changes accompanying PBO administration are the same as changes accompanying P4 administration. The between‐group comparison of such GM ratios (i.e., the change attributable to progesterone administration in the control group vs. the change attributable to progesterone administration in the PCOS group) is also expressed as a ratio of ratios with the accompanying 95% confidence interval; the unadjusted *p* value (and the Bonferroni‐adjusted *p* value in brackets) is for the null hypothesis test that changes attributable to progesterone in the control group are the same as changes attributable to progesterone in the PCOS group

Simple summary statistics for mean FSH are shown in Table 3 (more detail is provided in supplemental materials), and intervention‐related changes are graphically represented in Figures 4 and 5. Pre‐intervention mean FSH was similar between the progesterone and placebo admissions in both groups (*p* > 0.2 for both). In controls, 10‐h GM mean FSH increased by 31% (95% CI, 12–54%; *p* = 0.001) and 97% (95% CI, 68–131%; *p* < 0.001) with placebo and progesterone administration, respectively, with a significantly greater increase with progesterone administration (ratio of GM ratios 1.50 [95% CI, 1.20–1.88]; *p* = 0.001). In women with PCOS, 10‐h GM mean FSH did not change significantly with placebo (16% increase [95% CI, −1–37%]; *p* = 0.063), but FSH increased 52% (95% CI, 29–78%; *p* < 0.001) with progesterone administration. The progesterone‐related increase in mean FSH appeared to exceed the placebo‐related increase in PCOS (ratio of GM ratios 1.30 [95% CI 1.04–1.63]; *p* = 0.024), although this difference was not significant after Bonferroni correction (*p* = 0.072). The changes in 10‐h mean FSH attributable to progesterone were similar between control and PCOS groups (ratio of ratios 0.87 [95% CI, 0.63–1.19]; *p* = 0.369).

FSH AUC results (Table 3 and supplemental materials) were generally similar to those for mean FSH. Controls exhibited a significantly greater increase in FSH AUC with progesterone versus placebo administration (ratio of GM ratios 1.53 [95% CI, 1.21–1.93]; *p* = 0.001), as did women with PCOS (ratio of GM ratios 1.35 [95% CI, 1.07–1.71]; *p* = 0.014). Changes in FSH AUC attributable to progesterone were similar between the control and PCOS groups (ratio of ratios 0.89 [95% CI, 0.64–1.24]; *p* = 0.461).

Taken together, these results suggest that progesterone acutely augments mean FSH and FSH AUC in E2‐pretreated women, but they do not support the hypothesis that acute progesterone augmentation of mean FSH or FSH AUC is impaired in women with PCOS.

### 3.7 Post hoc sensitivity analyses

Since group differences in achieved progesterone and estradiol concentrations could have been confounding factors, analyses were repeated while simultaneously adjusting for differences in achieved progesterone and estradiol concentrations. Overall, this covariate‐adjusted analysis did not materially change the results (see supplemental materials). In summary, the changes in 10‐h LH pulse frequency, mean LH, LH AUC, pulsatile LH secretion, basal LH secretion, LH half‐life, mean FSH, and FSH AUC attributable to progesterone were similar between the control and PCOS groups after adjusting for differences in achieved progesterone and estradiol concentrations. The changes in 10‐h LH pulse mass attributable to progesterone appeared less pronounced in women with PCOS compared to controls after adjusting for these covariates (ratio of ratios, 0.46 [95% CI, 0.22–0.99]; uncorrected *p* = 0.047), but this was not significant after Bonferroni‐correction (*p* = 0.094).

As explained in Statistical analysis, analyses were repeated after excluding one subject in the control group. While the results of several of these sensitivity analyses did not materially alter interpretation, the results of some were different in potentially important ways (see supplemental materials). In particular, after excluding the one control subject, the following progesterone‐related changes were less prominent in PCOS compared to controls: progesterone concentration (ratio of ratios 0.59 [95% CI, 0.40–0.88]; Bonferroni‐corrected *p* = 0.034); LH pulse mass (ratio of ratios 0.36 [95% CI, 0.18–0.73]; Bonferroni‐corrected *p* = 0.013); pulsatile LH secretion (ratio of ratios 0.39 [95% CI, 0.18–0.86]; Bonferroni‐corrected *p* = 0.045); and mean FSH (ratio of ratios 0.78 [95% CI, 0.63–0.95]; Bonferroni‐corrected *p* = 0.050). After excluding the one control subject, the following progesterone‐related changes trended lower in PCOS compared to controls: LH AUC (ratio of ratios 0.53 [95% CI, 0.29–0.96]; uncorrected *p* = 0.039, Bonferroni‐corrected *p* = 0.078); and FSH AUC (ratio of ratios 0.79 [95% CI, 0.64–0.97], uncorrected *p* = 0.028, Bonferroni‐corrected *p* = 0.084).

## 4 DISCUSSION

The primary purpose of this study was to test the hypothesis that progesterone administration acutely reduces LH pulse frequency in normally cycling controls, but to a lesser degree in PCOS. However, exogenous progesterone did not acutely lower (within 12 h) LH pulse frequency in controls in this study (Hutchens et al., 2016). Similarly, exogenous progesterone did not acutely suppress LH pulse frequency in PCOS, and there was no difference in the degree of LH pulse frequency suppression between controls and PCOS. Thus, the results of this study did not support the primary hypothesis.

The rapidity with which progesterone reduces LH pulse frequency in normal women—whether over hours or days—remains unknown. Permezel et al. reported that, in 4 normally cycling women, 10 mg intramuscular progesterone (yielding mean progesterone concentrations of 1.6 ng/ml) lowered LH pulse frequency within 8 h (*p* = 0.05) during the follicular phase (Permezel et al., 1989). Another study suggested that LH pulse frequency decreased by about 50% in normal women (*n* = 5) within 5 days of exogenous progesterone (vaginal 50 mg every 8 h) and estradiol (transdermal 0.2 mg/day) during late follicular phase (Pastor et al., 1998). This apparent difference was not statistically significant, however, perhaps due to the small number of subjects studied.

In contrast to the current results in normally cycling women, previous studies suggested that exogenous progesterone acutely reduces waking LH pulse frequency within 3–6 h in early pubertal girls and within 12–16 h in late pubertal girls (Collins et al., 2012; Kim et al., 2018). These apparently discordant findings may possibly reflect the ability of androgens to impact both the degree and rapidity of progesterone negative feedback on LH pulse frequency. In particular, the negative‐feedback effects of progesterone on LH pulse frequency may be more rapid during puberty when androgen concentrations are lower. In contrast, when androgen concentrations are higher, as in adult women, LH pulse frequency reduction may require a longer duration of progesterone exposure.

An ability to suppress GnRH pulse frequency appears to be important for normal cyclic function (Lam & Ferin, 1987; Soules et al., 1987). In PCOS, the GnRH pulse generator is relatively resistant to the negative feedback effects of longer‐term progesterone exposure (Blank et al., 2009; Daniels & Berga, 1997; Pastor et al., 1998). Pastor et al. reported that LH pulse frequency was reduced by 60% in controls, but by only 25% in PCOS, after receiving exogenous progesterone and estradiol for 7 days (Pastor et al., 1998). This relative resistance to progesterone negative feedback appears to be mediated by testosterone excess. For instance, sensitivity to progesterone negative feedback was restored in women with PCOS after flutamide (androgen‐receptor blocker) administration for 4 weeks (Eagleson et al., 2000). A number of animal studies also suggest that androgen excess impairs progesterone negative feedback on the GnRH pulse generator (McCartney & Campbell, 2020). In this study, 12 h of progesterone exposure may have been insufficient for progesterone to have its full negative feedback effect on the GnRH pulse generator, and it is possible that a longer duration of progesterone (e.g., 24–72 h of exposure) might disclose differences in the rapidity of progesterone‐related suppression of LH pulse frequency in PCOS vs. controls.

An a priori secondary hypothesis was that exogenous progesterone acutely increases LH and FSH release more so in normally cycling women compared to women with PCOS. That is, in addition to relative resistance to the negative feedback actions of progesterone on LH (GnRH) pulse frequency, women with PCOS may demonstrate relative resistance to the *positive* feedback effects of progesterone on gonadotropin release. If correct, this could represent another mechanism for ovulatory dysfunction in PCOS. In adult women, high mid‐cycle estradiol concentrations (>200–300 pg/ml) trigger the gonadotropin surge—characterized by an approximately 10‐fold increase in LH and 4‐fold increase in FSH levels (Hall, 2018). In women, this phenomenon primarily appears to reflect an increase in pituitary gonadotropin responsiveness to GnRH release (Hall, 2018). As described above, the pre‐ovulatory increase in circulating progesterone may augment estradiol positive feedback at mid‐cycle (Chang & Jaffe, 1978; Liu & Yen, 1983), and some studies suggest that progesterone positive feedback is important for normal surge characteristics (e.g., surge latency period, surge duration, and FSH surge expression) (Chang & Jaffe, 1978; Liu & Yen, 1983; March et al., 1979; Taylor et al., 1995).

In this study, mean serum LH and FSH concentrations acutely increased with progesterone administration in both controls and PCOS. However, this study did not formally support the hypothesis that progesterone‐mediated augmentation of gonadotropin concentrations is impaired in PCOS. That is, while these data suggest that acute progesterone‐related augmentation of LH pulse mass and pulsatile LH secretion may be impaired in PCOS, such differences were not statistically significant after applying the highly conservative Bonferroni method of correcting for multiple comparisons. Importantly, this study was not powered to detect differences in secondary outcomes such as LH pulse mass and pulsatile LH secretion. Also of importance, the PCOS group also had higher BMI and body fat percentage (Table 1), and adiposity represents an important potential confounder with regard to factors such as average circulating LH concentrations and LH pulse amplitude (Arroyo et al., 1997; Morales et al., 1996; Pagan et al., 2006; Taylor et al., 1997).

The possibility of impaired estrogen positive feedback and gonadotropin surge generation in PCOS is not well studied. In prenatally androgenized female rats and sheep—animal models with PCOS‐like features—high‐dose estrogen does not induce gonadotropin surges (Foecking et al., 2005; Moore et al., 2013; Sharma et al., 2002; Unsworth et al., 2005). One study in women reported that the LH peak magnitude was similar in both PCOS and control groups after 3 days of high‐dose oral ethinyl estradiol (Baird et al., 1977). However, this study had several limitations including a non‐physiological method of estrogen delivery (ethinyl estradiol, 200 mcg/day), the use of daily blood samples, and the study of control subjects during the early follicular phase. The possibility of impaired estrogen positive feedback on gonadotropin release—and thus impaired gonadotropin surge generation—in PCOS deserves further study.

In conclusion, this study suggested that LH pulse frequency is not suppressed within 12 h after oral exogenous progesterone administration (100 mg) in estradiol‐pretreated women, regardless of PCOS status. Progesterone acutely increased various aspects of LH and FSH release in women with and without PCOS. Although this study suggests that progesterone‐mediated augmentation of LH pulse mass and pulsatile LH secretion may be impaired in PCOS compared to normally cycling women, such differences were not formally demonstrable.

## CONFLICT OF INTEREST

The authors have no conflicts of interest to disclose.

## AUTHOR CONTRIBUTIONS

C.R.M. conceived and designed the research study. C.R.M., S.H.K., C.M.B.S., and J.A.L. provided oversight to screening and study admission procedures. JTP, CRM, and SHK performed data analysis. C.R.M., S.H.K., and J.T.P. interpreted the results. S.H.K., C.R.M., and J.T.P. wrote the manuscript. C.R.M., S.H.K., and J.T.P. prepared tables and figures. C.M.B.S. and J.A.L. reviewed and provided editorial input into the manuscript. All authors approved the final version of the manuscript.
